# Supplementary material for: A Dansyl-Modified Sphingosine Kinase Inhibitor DPF-543 Enhanced De Novo Ceramide Generation
Source: Int J Mol Sci. 2021 Aug 25;22(17):9190. doi: 10.3390/ijms22179190 (PMC8431253; doi:10.3390/ijms22179190)
Supplement: Supplementary file 1 [file ijms-22-09190-s001.zip › ijms-1347256-supplementary.pdf]

Supplementary Data

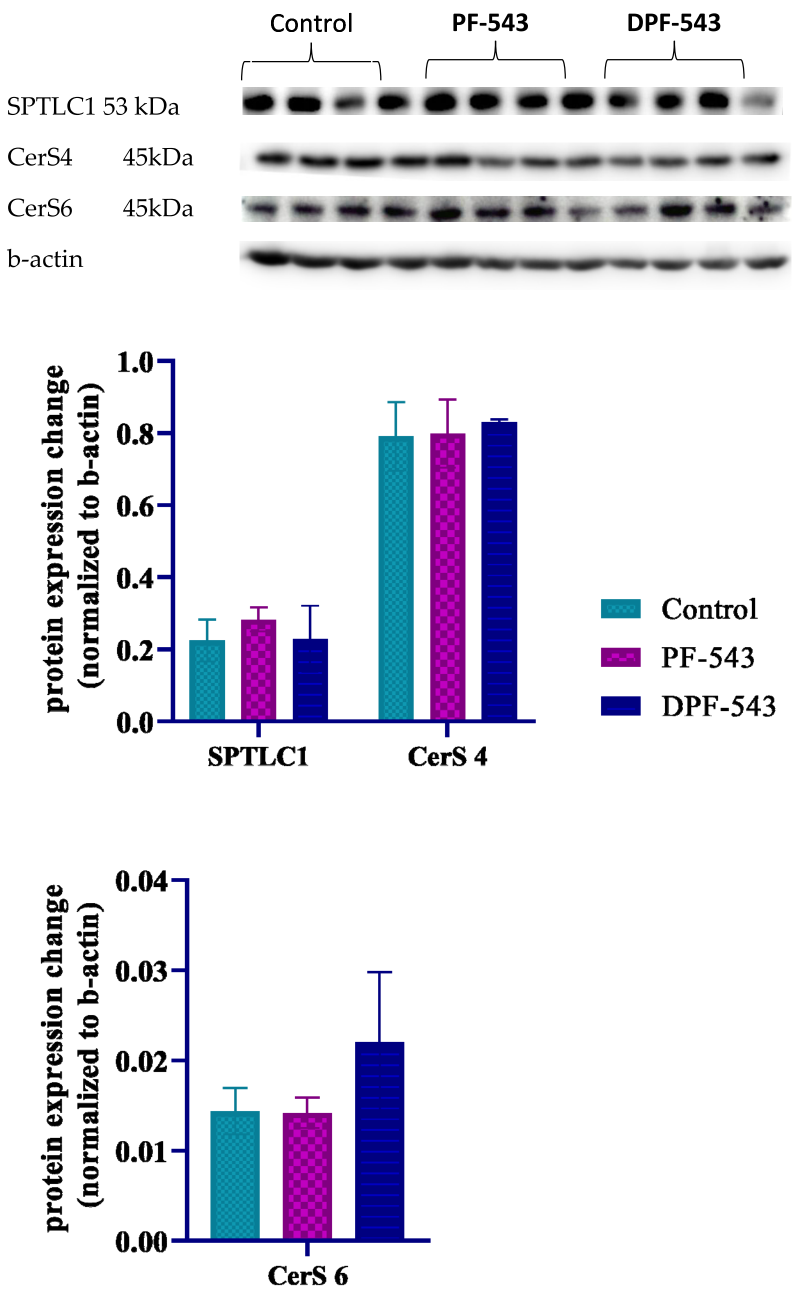

Figure S1

Table S1. QTRAP 4500 mass spectrometer setting for C18:1 Ceramide analysis

ID	Q1 (Da)	Q3 (Da)	Dwell time (ms)	DP(volts)	EP(volts)	CE(volts)	CXP(volts)
Cer 16:0	538.3	264.3	50	40	5	35	15
Cer 17:0	552.2	264.3	50	50	5	35	8
Cer 18:1	564.3	264.3	50	50	5	35	15
Cer 18:0	566.3	264.3	50	50	5.5	35	10
Cer 20:0	594.3	264.3	50	55	5	35	15
Cer 24:1	648.4	264.3	50	55	6.25	35	15

Cer 24:0	650.4	264.3	50	55	6.25	35	15

Table S2. QTRAP 4500 mass spectrometer setting for C17-DHCer and C17-Cer analysis.

ID	Q1 (Da)	Q3 (Da)	Dwell time (ms)	DP(volts)	EP(volts)	CE(volts)	CXP(volts)
Cer 16:0	524.3	250.3	50	40	5	35	15
Cer 17:0	552.2	264.3	50	50	5	35	8
Cer 18:1	550.4	250.3	50	50	5	35	15
Cer 18:0	552.4	250.3	50	50	5.5	35	10
Cer 20:0	580.4	250.3	50	55	5	35	15
Cer 22:0	608.6	250.3	50	55	5	35	15
Cer 24:1	634.4	250.3	50	55	6.25	35	15
Cer 24:0	636.4	250.3	50	55	6.25	35	15
DHCer 16:0	526.4	252.3	50	50	5.5	35	15
DHCer 18:1	550.4	252.3	50	50	5	35	14
DHCer 18:0	552.4	252.3	50	50	5	35	16
DHCer 20:0	582.4	252.3	50	50	5	35	15
DHCer 22:0	610.6	252.3	50	50	5	35	15
DHCer 24:1	636.4	252.3	50	50	6.5	35	15
DHCer 24:0	638.4	252.3	50	50	6.5	35	15

Table S3. QTRAP 4500 mass spectrometer setting for labeled and endogenous sphingolipid analysis

ID	Q1 (Da)	Q3 (Da)	Dwell time (ms)	DP(volts)	EP(volts)	CE(volts)	CXP(volts)
KDS-D2	302.2	272.3	150	35	5	35	15
SPA-D2	304.3	286.3	150	35	5	35	15
KDS	300.2	270.3	150	35	5	35	15
SPA	302.3	284.3	150	35	5	35	15
SPH	300.3	282.3	150	35	5	35	15
C17-SPH	286.3	268.3	150	35	5	35	15

Table S4. C17-DHCer conversion rate to C17-Cer in percentage

Groups/ Ceramides	C16:0	C18:1	C18:0	C20:0	C22:0	C24:1	C24:0
Control	0.78	0.018	0.47	7.7	53.1	43.8	27.5
PF-543	1.92	0.018	1.30	22	110.2	40.5	21.3
DPF-543	1.84	0.019	1.02	19.6	268.3	78.5	14.9
